# Nicotinism vs. Glomerulopathies—Smoking as a Risk Factor for Primary Glomerulopathies

**DOI:** 10.3390/antiox14101233

**Published:** 2025-10-14

**Authors:** Magdalena Dzięgiel, Aleksandra Maciejowska, Marek Misiak, Katarzyna A. Lisowska

**Affiliations:** 1 Student Scientific Club, Department of Physiopathology, Medical University of Gdańsk, 80-211 Gdańsk, Poland; amaciejowska@gumed.edu.pl (A.M.); misiak.m@student.uksw.edu.pl (M.M.); 2 Department of Rheumatology, Clinical Immunology, Geriatrics and Internal Medicine, Medical University of Gdańsk, 80-214 Gdańsk, Poland

**Keywords:** nicotine, glomerulopathy, IgA nephropathy, minimal change disease, membranous nephropathy, tobacco smoking, cigarette smoking, kidney

## Abstract

Smoking cigarettes affects the human body on many levels—not only are the lungs and heart targeted, but also other organs, directly and through derived alterations. We decided to parallel the impacts most often described in the literature in the hope of better future targeting regarding treatment for smoke-induced renal injury. As a result of our research, it is clear that damage is mostly localized directly in vessels and glomeruli. We perceive it as a connected web, where oxidative stress leads to local inflammation, general inflammation in the form of obesity, or inflammation due to nasopharyngeal infection. It later affects other types of tissues: podocytes, epithelium in both glomeruli, renal tubules, and vessels. We mention major molecules proven to participate in kidney damage that tend to be similar in all disease entities depicted in this study: IgA nephropathy, membranous nephropathy, and minimal change disease. Moreover, as nicotine is a major component of both classic cigarettes and electronic cigarettes, we decided to approximate and summarize the information on its impact on primary glomerulopathies.

## 1. Introduction

Approximately 1.3 billion people worldwide are cigarette smokers [1,2], and these figures are expected to rise in the coming decades [3]. At the same time, 4.5 million students admit to using tobacco products [4]. Data reported in *The Lancet Public Health* (2021) indicate that the majority of smokers—approximately 82.6%—initiated smoking between the ages of 14 and 25, with nearly one in five (18.5%) becoming regular smokers before turning 15 [5].

While smoking prevalence has decreased notably since 1990 in adults aged 15 years and older, the absolute number of smokers has nevertheless increased, driven by global population growth, from about 0.99 billion in 1990 [3] to about 1.3 billion tobacco users nowadays [2]. Tobacco exposure is a known risk factor for cardiovascular disease (CVD) [6], coronary heart disease (CHD), hypertension, cardiopulmonary dysfunction, myocardial infarction [7,8,9,10,11], and progression of kidney disease [12,13,14,15]. There is still uncertainty about whether every kidney disease is equally vulnerable due to cigarette smoking [16]. However, clinical observations suggest that there is a high likelihood that exposure to tobacco smoke may promote the progression of chronic kidney disease (CKD). For example, children exposed to cigarette smoke have higher systolic blood pressure, daytime blood pressure load, and blood pressure variability compared to children without that kind of exposure [17]. Some studies associate exposure to tobacco smoke with proteinuria [18,19]. Tobacco exposure is also connected with other physiological dysfunctions, e.g., cancer, atherosclerosis, thrombogenesis, and vascular occlusion [20,21,22,23,24].

Even though multiple studies have linked tobacco exposure to CKD, the direct link between smoking and proteinuric glomerulopathies has not been well described. Cigarette smoking is expected to cause approximately 8 million mortalities globally every year, including more than a million non-smokers exposed to second-hand smoke [2]. Smoking stimulates the sympathetic nervous system, alters endothelial activity, induces smooth muscle cell proliferation, and causes dysfunction of vascular tone regulators, which can lead to the onset and progression of arterial damage [25,26].

CKD, characterized by a declining estimated glomerular filtration rate (eGFR) and albuminuria, has become a serious public health issue in recent years, affecting the health of the population [27]. An estimated 10–15% of people worldwide have CKD, making it a serious public health burden [28]. Risk factors for CKD include hypertension [29], diabetes mellitus [30], overweight [31], COVID-19 [32], nephrotoxic drugs, aging [33], and air pollution exposure [34].

Tobacco exposure is recognized as a risk factor for progression of chronic kidney disease and proteinuria in children and adults [18,19,35]. Some reports show that the ratio of nicotine metabolites is negatively correlated with kidney function in adults [36]. Nicotine and its metabolites are known to be directly cytotoxic to glomerular visceral epithelial cells (i.e., podocytes), leading to proteinuria and progression of CKD [37,38,39]. Glomerular disease may also occur secondary to other causes, including autoimmune diseases, infections, medications, or malignancies [40]. In the literature, examples of alleged correlation between cigarette smoke and progression of primary glomerulopathies are shown in individual studies, with a lack of explanation of the exact mechanism of action on their development.

Although the detrimental impact of smoking on cancer and cardiovascular disease is well established, its role in renal pathology, specifically glomerular injury and the progression of chronic kidney disease, is less widely recognized. According to the popularity of tobacco use, there is a need to study the correlation between components of tobacco smoke and other conditions, such as glomerulopathies.

This review aims to synthesize current knowledge on the impact of smoking and nicotine on kidney diseases, in particular, various types of primary glomerulopathies, including minimal change disease (MCD), IgA nephropathy, and membranous nephropathy (MN). This review focused on how cigarette smoke is a risk factor for glomerulopathies.

## 2. Methods

A systematic literature review of the link between smoking and various types of glomerulopathies (GN) is presented. A total of 196 papers published between 2020 and 2025 were critically assessed and included in the manuscript. The following Medical Subject Headings terms were used: nephropathy IgA, smoking (PubMed; 16 documents found), nephropathy AND IgA AND smoking OR tobacco OR nicotine (Scopus; 36 documents found), membranous nephropathy AND smoking (PubMed 11, Scopus 40 documents found), minimal change disease AND minimal change nephropathy AND smoking (PubMed 6, Scopus 9 documents found). Prespecified inclusion and exclusion criteria in our research protocol determined whether studies were eligible for full-text analysis. Three reviewers (M.D., A.M., and M.M.) independently screened all manuscripts for relevance to the chosen inclusion criteria. Full-text publications were assessed for definitive inclusion independently by all authors.

## 3. Glomerulonephritis

The nephron is the foundation of kidney structure. It consists, in general, of glomeruli and tubules. In the glomerulus, the main process is primary filtration, which leads to the creation of primary urine. Bowman’s capsule—outer garment of glomerulus—is layered from the inside with glomerular basement membrane (GBM) and with visceral epithelial cells, podocytes, and mesangial cells on the outside. Those comprise filtering barriers for plasma [41].

Glomerulonephritis encompasses a heterogeneous group of renal diseases characterized by injury affecting various components of the glomerulus, including podocytes, slit diaphragms, mesangial cells, the GBM, and the capillary endothelium; in some cases, the tubulointerstitium may also be involved [42]. GN can manifest as a primary kidney disease limited to the glomeruli without evidence of systemic disease, in contrast to secondary GN, which reflects the renal involvement of systemic disorders such as systemic lupus erythematosus (SLE), monoclonal gammopathies, or diabetic nephropathy [43].

Traditionally, GN is classified based on histopathological findings from kidney biopsy, which consider factors such as the location and type of deposits, the degree of cellular proliferation, and fibrosis [44]. Within this system, the primary glomerulopathies include IgA nephropathy (IgAN), minimal change disease (MCD), focal segmental glomerulosclerosis (FSGS), primary membranous nephropathy (PMN), and primary membranoproliferative glomerulonephritis (MPGN) [45].

Advances in immunophenotyping and genetics have revealed that identical histological patterns can arise from distinct underlying diseases and due to different risk factors, each of which may need a different therapeutic approach [44]. Therefore, new classification systems for GN are currently being proposed. Anders et al. [42] and Romagnani et al. [44] suggested that GN requires a classification system based on an immunopathophysiology to better connect with the growing number of available immunomodulating drugs, highlighting five groups: infection-related, autoimmune, alloimmune, autoinflammatory, and monoclonal gammopathy-related GN. For this reason, we aimed to outline especially the underlying pathomechanisms and describe how smoking can influence these processes, and then indicate the specific primary glomerular diseases in which this effect has been observed.

Glomerulonephritis, although relatively rare, remains an important cause of chronic kidney disease (CKD) and end-stage renal disease (ESRD) worldwide. Its incidence varies between 0.2/100,000/year and 2.5/100,000/year [46]. According to most CKD registries, glomerular diseases represent roughly 20–25% of all prevalent cases [47,48], which makes it still a major cause of CKD [49]. In pediatric and young adult populations, glomerular disease frequently leads to irreversible renal damage [48]. GN accounts for approximately 10–25% of ESRD cases in Europe and the US, although its relative contribution varies by region, age, and ethnicity [41].

It is noteworthy that, over the last fifty years, there has been a substantial shift in the epidemiology of glomerular diseases worldwide [50]. Currently, it is well established that IgAN represents the most common and MN the second most common type of GN. Since the 1990s, MN has lost its position as the leading cause of nephrotic syndrome, which has been overtaken by focal segmental glomerulosclerosis (FSGS), the third common GN [50].

Primary GN may manifest as well-defined clinical glomerular syndromes, such as isolated urinary abnormalities, nephritic syndrome, nephrotic syndrome, rapidly progressive glomerulonephritis (RPGN), and chronic kidney disease, which can occasionally progress to ESRD demanding dialysis or transplantation [45]. The hallmark clinical feature is proteinuria, which can range from mild elevations to nephrotic-range levels, frequently accompanied by microscopic or macroscopic hematuria [48]. Additional clinical manifestations observed within these syndromes may include varying degrees of azotemia, hypertension, edema, oliguria, fatigue, headache, and malaise [41]. Hypertension is frequently present and can contribute to the progression of kidney injury [51]. Edema, which results from hypoalbuminemia and disordered fluid homeostasis, is particularly common in nephrotic presentations, often first appearing around the periorbital region or lower extremities, and may even be the earliest clinically apparent sign. In rapidly progressive forms, oliguria and acute kidney injury can develop, while systemic symptoms such as fatigue and general malaise tend to be more pronounced in advanced disease stages. Less commonly, hyperlipidemia and thrombotic disease may occur [52].

The severity and constellation of these features largely depend on the underlying histopathological subtype and the balance between disease activity and chronicity, reflecting the pathophysiological process. For instance, in children and young adults, nephrotic syndrome is more likely to be associated with MCD or FSGS; in contrast, in older adults, MN is a more typical cause. Meanwhile, IgAN often presents with episodic macroscopic hematuria, sometimes accompanied by mild edema or hypertension, typically following upper respiratory tract infections [47]. Renal biopsy is essential for confirming GN, assessing the level of ongoing immunologic injury, and evaluating the degree of established, irreversible structural damage [44].

Management of primary glomerulopathies typically combines optimal supportive care, such as rigorous blood pressure control, sodium restriction, and reductions in proteinuria, with disease-specific immunosuppressive or immunomodulatory therapy, tailored according to histological subtype, proteinuria severity, and progression risk as outlined in the Kidney Disease Improving Global Outcomes (KDIGO) 2021 Clinical Practice Guideline [48].

This study concentrates on IgA nephropathy (IgAN), minimal change disease (MCD), and membranous nephropathy (MN), as relatively accessible clinical and pathological data support these. Less common primary glomerulopathies are partially described due to sparse literature and lower prevalence.

### 3.1. IgA Nephropathy

IgA nephropathy, also called Berger disease [53], is the most common cause of all primary glomerulopathies worldwide [54,55]. IgA is the predominant immunoglobulin of mucosal surfaces, and activation of the mucosal immune system can trigger IgAN production. This activation might be an effect of upper respiratory tract or gastrointestinal infection, major mucous-layered surfaces in the human body [55]. Glomerular inflammation and mesangial proliferation occur due to nephritogenic immune complexes. Then activation of the renin–angiotensin–aldosterone (RAA) and complement system leads to glomerulosclerosis and tubulointerstitial fibrosis, which finally leads to impaired renal function. Risk factors, such as smoking and hypertension, exacerbate disease progression by causing microvascular injury. Glomerulomegaly and maladaptive hyperfiltration related to obesity may also be implicated in the nonimmunological progression of the disease [53].

There are at least four hits needed to induce IgA nephropathy. The initial two depend on increased circulating galactose-deficient IgA [55], which is due to abnormal O-glycosylation of the IgA1 hinge region and production of unique anti-glycan and anti-IgA1 antibodies [45,53]. The first hit is associated with a genetic predisposition to developing a dysregulated immune response [53]. In genetically susceptible people, a precipitating factor, such as infection, can trigger B cells to produce IgA1 missing a galactose molecule at the hinge region. Mucosal infections, chronic exposure to pathogens, or improper handling of gut commensals can trigger the abnormal immune response [53]. Finally, hypoglycosylation enables the aberrant IgA1 molecules to aggregate.

The hallmark is IgA deposition in glomerular mesangium with associated mesangial expansion and/or hypercellularity [45]. The Oxford Classification is used to score the histology according to the degree of mesangial, endocapillary, segmental, and tubule-interstitial involvement, as well as the presence of glomerular crescents [45].

Genetically, the presence of hypoglycosylated IgA1 is a heritable trait. In one fourth of blood relatives of IgAN patients, serum galactose-deficient IgA1 levels are elevated [55]. When it comes to treatment, KDIGO Guidelines for Glomerulonephritis management should focus on best supportive care, reducing proteinuria with the RAA system inhibition, and optimizing blood pressure control [45].

### 3.2. Membranous Nephropathy

Membranous nephropathy (MN), also known as membranous glomerulopathy, is characterized by the subepithelial deposition of immune complexes (ICs) along the glomerular basement membrane, resulting in diffuse GBM thickening and progressive podocyte injury [56,57]. Histopathologically, MN is distinguished by the presence of granular IgG and complement component C3 deposits along the GBM, manifesting as a strong, finely granular pattern on immunofluorescence microscopy [58]. Electron microscopy reveals electron-dense deposits localized in the subepithelial space, often associated with characteristic basement membrane spikes.

MN is the second most common glomerulopathy after IgAN [59]. It accounts for approximately 30% of nephrotic syndrome cases in adults and represents the leading cause of nephrotic syndrome among white adults [60] and the second worldwide. The global incidence of MN is approximately 1.2 cases per 100,000 annually, predominantly affecting middle-aged and older adults but also occurring, less frequently, in children [46]. Progressing to nephrotic syndrome in approximately 80% of cases [61] and eventually to ESRD in roughly one-third of patients if proteinuria remains uncontrolled.

The first experimental model that provided insights into the disease pathophysiology was developed by Heymann et al. in 1959, who demonstrated in rats that MN could arise from the in situ formation of ICs generated by antibodies directed against antigens located beneath the podocytes [62]. Since then, considerable progress has been made in understanding the underlying mechanisms of MN, particularly with the identification of major autoantigens [59]. Advances in immunology have revealed circulating autoantibodies that recognize several podocyte antigens, including the phospholipase A2 receptor (PLA2R), accounting for >70% of cases [63], thrombospondin type-1 domain-containing 7A (THSD7A) present in 2–5% of MN patients [64], and, more recently, neural epidermal growth factor-like 1 (NELL1). To date, more than 14 target antigens have been identified, collectively explaining approximately 80–90% of MN cases. These discoveries have helped redefine MN as an organ-specific autoimmune disease [65].

MN is traditionally classified into primary (PMN) or idiopathic MN (iMN), which occurs in genetically predisposed individuals, and secondary MN, associated with known etiologies such as infections (hepatitis B and C, HIV, SARS-CoV-2), autoimmune diseases (SLE, rheumatoid arthritis), malignancies, and drug/toxin exposures [65]. Approximately 70–75% of adult cases of MN are classified as PMN, which is characterized by the binding of circulating autoantibodies, most notably of the IgG4 subclass, to podocyte antigens. Although the precise triggers of PMN remain incompletely understood, observational studies suggest potential links with genetic predisposition, infections, and environmental exposures, including components derived from tobacco smoke. Ponticelli et al. [58] indicated that undetected viral infections or ecological pollutants may initiate PMN by exposing podocyte antigens in genetically susceptible individuals, thereby provoking an autoimmune response.

### 3.3. Minimal Change Disease

Minimal change disease (MCD), or minimal change nephropathy (MCN), is defined as diffuse podocytopathy with nearly normal findings on light microscopy (LM), contrasted by striking podocyte damage visible on electron microscopy (EM), including diffuse effacement of foot processes and disappearance of slit diaphragms, yet notably lacking electron-dense deposits [66]. The GBM is morphologically unremarkable but has decreased charge, which was believed to be responsible for the development of selective proteinuria in MCD [67].

Significantly, MCD represents the leading cause of nephrotic syndrome in the pediatric population, responsible for about 70–90% of cases, and remains an important cause among adults, where it accounts for approximately 10–25% [68]. The cause of MCD is unknown, but many authors believe that immunologic dysregulation and modifications of the podocyte synergize in altering the GBM integrity. It has been suggested that aberrant immune response against podocytes is caused by the T-cell compartment [69]. As to changes in podocyte structure, the foot process effacement is visible by electron microscopy. The research was focused on reasons for disrupted integrity of the glomerular filtration barrier [66].

Some authors implicated that changes in the regulation of the T-cell function are responsible for driving podocyte injury in MCD [70]. Although some abnormalities of T-cell subsets and their activity in vitro have been described, the literature contains many contradictions. These abnormalities are also suggested to be secondary to the nephrotic state rather than primary [71], as MCD has been observed in immune dysregulation, polyendocrinopathy, enteropathy, X-linked syndrome (IPEX), a congenital immunodeficiency with severe T regulatory cell (Treg) hypofunction [72].

Meanwhile, animal models and observations in humans suggest that reactive oxygen species (ROS) could also be involved in the pathogenesis of MCN. ROS are first-phase mediators produced by polymorphonuclear cells (PMNs) in response to infectious triggers. Bertelli et al. [73] first demonstrated that PMNs can produce high quantities of ROS in children with MCN during relapses of proteinuria, which they associated with a lack of a negative regulatory circuit that involves anti-inflammatory soluble factors derived from Tregs. Also, oxidant production was negatively correlated with the amount of Treg expression of CD39 antigen [73]—an enzyme in the conversion of ATP to immunomodulatory adenosine, present, among others, in endothelial cells [74]. Indirect evidence for oxidant activity in vivo comes from the observation that a significant part of serum albumin is oxidized in MCN patients. Albumin has only recently been recognized as the major antioxidant in serum that plays a crucial role during infections in humans. Musante et al. [75] characterized serum albumin in children with MCN employing mass spectrometry, and showed chemical modifications of the unique free cysteine residue of the protein sequence to a sulfonic acid (Cys-SO_3_H) that is the end product of its oxidation, and therefore represents a surrogate biomarker of oxidative stress [76].

## 4. Cigarette Smoking Versus the Occurrence of Primary Glomerulopathies

Given the notable prevalence of GN and the widespread exposure to tobacco smoking globally, exploring the potential association is warranted. However, precise incidence data of GN among smokers are insufficient, as GN is a relatively rare disease, and population-based registers rarely capture smoking status. In the NEPTUNE cohort, 371 adults and 192 children with proteinuric glomerulonephritis were recruited. At baseline, 14.6% of adults were active smokers, 29.1% were former smokers, and 4.9% reported passive exposure. Among children, active smoking was rare (0,5%), but 16.7% of children patients reported passive exposure to tobacco smoke. These findings underscore that nearly half of adult GN patients had a history of active smoking [77], compared with ~20% of adults in the general US population [4]. In contrast, Stengel et al. [78] found that the overall prevalence of smoking among adult men with GN was similar to that of hospital controls (60% vs. 65%), indicating no clear overrepresentation of smokers in GN in that cohort. The authors included 295 adult patients with biopsy-proven primary GN cases (including MN, IgAN, MCD, and FSGS) and 242 matched hospital controls. They demonstrated a significant association between cigarette smoking and the severity of primary GN, reflected by a higher prevalence of chronic renal failure (CRF) among male smokers, particularly those over 40 years of age and with hypertension. A clear dose–response relationship was observed, with greater daily consumption and cumulative exposure correlating with an increased risk of CRF. Interestingly, former smokers showed a higher risk than current smokers, likely due to longer exposure. The effect of smoking was modified by age and hypertension, suggesting its role in disease progression among high-risk male patients. Although causality cannot be confirmed, these findings strengthen the evidence for a potential pathogenic role of smoking in GN. Notably, there was no significant association found in women between smoking and CRF and with the incidence of specific histological GN subtypes.

This section explores the study-proven role of smoking in GN, summarizing clinical data and experimental findings that elucidate its potential impact on disease development and outcomes.

### 4.1. IgA Nephropathy

Both general research and case reports of patients inform about the noticeable link between the presence of smoking and worsening of the course of IgA nephropathy in already diagnosed people. For example, Wang et al. [79] studied, apart from non-smokers, current and former smokers with primary glomerulonephritis. The indicators that were examined were hypertension (%), systolic blood pressure (SBP) (mmHg), diastolic blood pressure (DBP) (mmHg), serum creatinine (µmol/L), estimated glomerular filtration rate (eGFR) (mL/min per 1.73 m^2^), and urine protein (g/24 h). As results showed, IgAN patients with a history of smoking were more likely to have hypertension and renal vascular changes, as well as worse renal outcomes, compared with patients without hypertension who did not smoke [79]. Moreover, current smoking was an independent risk factor for progression of micro- or macroalbuminuria and ESRD among patients with diabetic nephropathy (DN) [79].

In another study, not only was smoking observed solely, but also its possible debuff with hyperuricemia [80]. The authors aimed to identify an association between smoking status and hyperuricemia and renal arteriolar hyalinosis and wall thickening in IgAN patients. Checked indicators were retrieved from renal biopsy and evaluated as grade 0, no hyalinosis of the vessel wall; grade 1, hyalinosis of <25%; grade 2, hyalinosis of 25–50%; and grade 3, hyalinosis of >50% of the vessel wall circumference. The scale used in this study highlights various factors considered to determine the state of the kidneys. The authors showed that smoking and hyperuricemia, but not smoking alone, were significantly associated with an increased risk of higher-grade renal arteriolar wall thickening. Another study showed that male patients with IgAN have more severe renal dysfunction compared with female patients, and smoking is an independent risk factor for crescent formation in the kidneys only in male patients [81].

### 4.2. Minimal Change Disease

Although many studies were based on MCD patents, very few considered the effect of nicotine on those patients, which brings quite poor literature covering both factors together. In the research by Zhang et al. [82], MCD patients had higher urine protein, serum creatinine, and cholesterol, and a lower serum albumin compared to healthy people. Seven of those patients were ex-smokers who had stopped smoking for 1 to 5 years.

### 4.3. Membranous Nephropathy

A multicenter cohort study including patients with PMN examined the impact of cigarette smoking on disease progression. Yamaguchi et al. [83] found that current smoking, higher daily cigarette consumption, and heavy cumulative exposure (≥40 pack-years) were all significantly associated with a 30% decline in eGFR. Notably, the risk increased in a clear dose-dependent manner, and older age and female sex further heightened susceptibility. However, smoking was not associated with achieving complete remission of proteinuria. These findings suggest that cigarette smoking is an important and modifiable risk factor that accelerates renal function decline in PMN [83].

In a retrospective single-center study from China, Jiang et al. [84] compared iMN (PMN) with an increasing number of atypical membranous nephropathy (aMN) cases of unknown etiology. Patients with aMN were significantly younger and had a notably higher prevalence of smoking (37.1% vs. 27%). Although nephrotic syndrome was the main clinical presentation in both groups, aMN patients more frequently also exhibited features of nephritic syndrome. Interestingly, serum anti-PLA2R antibody levels did not distinguish between aMN and iMN. The authors suggest that aMN may represent a distinct clinical and pathological entity, potentially influenced by environmental factors such as smoking, warranting further investigation on a larger scale. Their study highlights the potential role of environmental factors such as smoking in the onset and progression of MN [84].

In a recent study applying deep learning to biopsy slides from patients with PMN, smoking emerged as an independent predictor of poorer response to immunosuppressive therapy. The model demonstrated that patients who smoked had a significantly reduced likelihood of achieving remission, highlighting smoking not only as a potential risk factor for disease progression but also as a modifier of treatment efficacy. Findings of Wei et al. [85] reinforce the clinical importance of smoking cessation in the management of iMN.

## 5. Proposed Mechanisms of Cigarette-Induced Kidney Damage

These days, it is suspected that smoking affects kidneys in many ways. Here, the most described in the literature are presented. It is evident that damage is primarily localized in vessels and glomeruli, resulting from oxidative stress that leads to local immunological disorders. These disorders subsequently affect various tissues, including podocytes and the epithelium in both glomeruli and vessels, potentially leading to hypertension and other complications. This topic should be considered in future research.

### 5.1. Role of Oxidative Stress

One of the most considered mechanisms of cigarette smoke in the causation of glomerulonephritis is reactive oxygen species (ROS). It was confirmed that ROS were found in higher amounts in smoking patients than in non-smokers [86]. ROS are derivatives of molecular oxygen (O_2_), which can later lead to the creation of superoxide (O_2_•−), hydrogen peroxide (H_2_O_2_), hypochlorous acid (HOCl), and peroxynitrite/peroxynitrous acid (ONOO−/ONOOH) [87]. Those also participate in oxidative stress (Table 1). ROS have a broad action spectrum in organisms. For example, the occurrence of ferroptosis is related to increased production of ROS and other oxides. Ferroptosis is an important, newly discovered form of cell death caused by the deficiency of glutathione peroxidase 4 (GPX4) and associated with oxidation [88].

**Figure 1:**
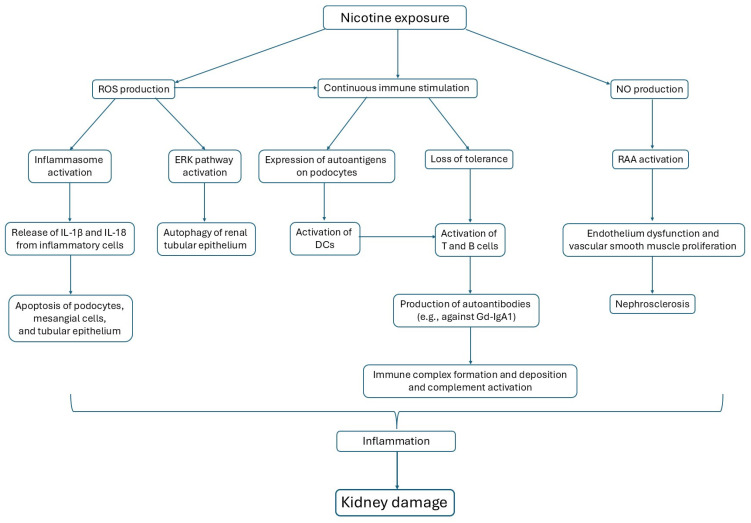
Three main pathways are activated by nicotine exposure, affecting glomerular cells.

Inflammasomes can be activated by pathogen-associated molecular patterns (PAMPs) or damage-associated molecular patterns (DAMPs) (Figure 1). The most typical mechanism for activating the inflammasome is the production of ROS. Inflammasome consists of NLRP3 (NOD-like receptor family pyrin domain containing 3), apoptosis-associated speck-like protein containing a caspase recruitment domain (ASC), and the pro-caspase-1 [88]. Activated by autocatalytic cleavage, caspase-1 then cleaves pro-interleukin-1β (pro-IL-1β) and pro-interleukin-18 (pro-IL-18) into their biologically active forms IL-1β and IL-18, causing inflammation and tissue damage. NLRP3 inflammasome itself is activated by a priming signal, such as ligands for Toll-like receptors (TLRs), NOD-like receptors (NLRs), or cytokine receptors, which initiate the transcription of nuclear factor-kappa B (NF-κB). NF-κB promotes the expression of NLRP3 and pro-IL-1β. Its activation is well described in the immune cells, but it might also happen in kidney cells, such as podocytes, mesangial cells, and renal tubular epithelium [89].

Studies have shown that the regulation of inflammasomes and autophagy could be the key to the treatment of multiple diseases, including kidney diseases [89]. Huang et al. [90] showed that cisplatin may trigger kidney injury by blocking autophagy and activating NLRP3 inflammasomes. The relationship between the inflammasome and autophagy can play a crucial role in the development of IgAN. In mouse models of progressive IgAN, resveratrol (a type of natural phenol or polyphenol produced by some plants in response to injury) inhibits NLRP3 inflammasome activation by augmenting autophagy and preserving mitochondrial integrity [91].

Based on model evidence, it was also found in induced accelerated progressive IgAN mice that luteolin, which is present in various plants, promoted the nuclear factor erythroid-derived 2-related factor 2 (Nrf2) antioxidant pathway [92]. Nrf2 is a transcription factor that stimulates the expression of antioxidants in response to oxidative damage. During oxidative stress, Nrf2 is transported to the nucleus, where it activates antioxidant response element (ARE)-dependent gene transcription to clear ROS and maintain cellular redox stability. Heme oxygenase-1 (HO-1) is a key antioxidant enzyme regulated by Nrf2. Some studies have confirmed that the upregulation of Nrf2 expression has a protective effect in IgAN animal models. Liang et al. [92] also demonstrated that luteolin significantly improved renal function by inhibiting NLRP3 inflammasome activation, reducing the concentration of pro-inflammatory cytokines and ROS. Based on these findings, it can be concluded that ROS inhibition may inhibit NLRP3 activation and reduce inflammation in IgAN [89]. In MN, inhibition of ROS reversed complement-induced pyroptosis in podocytes. ROS inhibitors also significantly reduce the expression of key protein molecules in the pyroptosis pathway, including NLRP3, ASC, pro-caspase-1, cleaved caspase-1, pro-IL-1β, and mature IL-1β [93].

As Deng et al. [94] state, in MCD, the ERK pathway in damaged renal tubular epithelial cells might be activated by a ROS-dependent mechanism. ERK, an extracellular signal-regulated protein kinase, is involved in various biological responses, such as cell differentiation and proliferation, cell morphology maintenance, cytoskeletal construction, and cell apoptosis. The ROS-ERK pathway can induce cellular injury and provide an autophagy-associated adaptive response [95]. In MN, a concept appears that a second wave of antibodies targeting superoxide dismutase 2 (SOD2) is generated in autoimmune glomerulonephritis and may negatively influence the clinical outcome. Explaining, knockout mice for SOD2 develop spontaneous proteinuria and focal glomerular damage [95]. Circulating and renal deposits of anti-SOD2 antibodies have been detected in MN patients. In MCD, it was suggested that ROS can modify the GBM selectivity versus circulating proteins by peroxidation of the lipid constituents of the membrane [95].

In IgAN, ROS, as well as chemical exposure to nicotine, may provoke the glomerular mesangial proliferation in the presence of aberrantly glycosylated IgA1 [96], targeting the kidneys directly. What we know is that cigarette smoke induces oxidative stress due to changes in cellular metabolism, which increases the stiffness of central vessels, raises general arterial pressure, and leads to tubular damage [79], associated with a loss of epithelial cells [97]. By contrast, smoking in MN induces oxidative stress and inflammation in the lungs, leading to increased serum levels of pro-inflammatory cytokines such as IL-6 and TNF-α (tumor necrosis factor-alpha), which may promote autoimmune responses, including autoantibody production and immune complex deposition in the kidneys [98]. This comparative data might emphasize different target regions, playing a crucial role in both diseases.

### 5.2. Role of Inflammation and Immunity

Another often noted impact of cigarette smoke on the kidneys is the augmented production of antibodies. Studies using animal models of MCD showed that a complex interaction of factors and cells exists, starting with activation of innate immunity and continuing with antigen presentation. For example, increased expression of lymphotoxin beta receptor (LTβR) ligands on innate immune cells enhances the NF-κB signaling and enriches LTβR-target gene expression, all in epithelial cells of lungs from mice exposed to chronic cigarette smoke [99]. CD80, responsible for the T-cell activation pathway, and CD40/CD40L antigens essential for the communication between T and B cells were found to be affected in tobacco-consuming oral cancer patients [100]. Distinctive studies propose the possibility that the same antigens are expressed by podocytes under inflammatory stimuli, representing a direct potential mechanism for proteinuria [101,102], as podocytes form the epithelial surface of the glomerulus, where filtration of molecules under 60 kDa takes place [103].

In MN, the most notable autoantigen is located on podocytes as well. For example, while the PLA2R antigen is primarily expressed in the kidney, lung inflammation may trigger or amplify autoimmune mechanisms contributing to MN pathogenesis, even without obvious lung symptoms. There was a case of a 77-year-old patient who suffered from PLA2R antibody-positive MN complicated with IgG4-related disease (IgG4-RD), only affecting the lung, who effectively responded to treatment for IgG4-RD [104]. In their retrospective investigation of 845 patients with IgG4-RD, Tsai et al. [105] suggested that smoking may precipitate the disease.

There are studies connecting kidney dysfunction to oral cavity disturbances in IgAN. Common knowledge is that cigarette smoke reaches tonsils and epipharynx, where nasopharynx-associated lymphoid tissue (NALT) and mucous tissue are present [106]. NALT is a mucosal-associated lymphoid organ implanted in the submucosa of the nasal passage, where inhaled antigens are deposited. During the steady state, conventional dendritic cells (cDCs) within the NALT suppress T-cell responses. [107]. However, in IgA, TLR9 (Toll-like receptor 9) activation in the NALT represents a common pathway by which exogenous antigens engage the mucosal immune system. TLR9 explicitly recognizes unmethylated DNA sequences in bacterial and viral DNA, thus leading to aggravation of renal injury in IgAN. Kano et al.’s study showed that stimulation of TLR9 with CpG oligonucleotides (CpG-ODN) enhanced the production of IgA in the supernatants of cells isolated from the NALT [108]. Moreover, it was shown that TLR9 polymorphisms are associated with disease progression in IgAN patients [109].

Continuous immune stimulation caused by smoking may contribute to the development of IgA nephropathy [106]. Chronic tonsillitis is frequently observed in IgAN patients. Moreover, in chronic tonsillitis, the expression of the J chain in IgA-positive plasma cells decreases in germinal centers (GC) and extrafollicular regions [106]. The J chain (encoded by the *JCHAIN* gene) regulates the polymerization of multimeric IgM and IgA by forming disulfide bonds with the C-termini of heavy chains, which facilitates their transport across mucosal epithelia [109]. In chronic tonsillitis, toxic bacterial factors may stimulate excessive antibody production, thereby contributing to the pathogenesis and progression of IgAN [105]. The exact mechanisms leading to the formation of anti-glycan antibodies are not fully understood. It is hypothesized that in IgAN patients, infection with bacteria expressing N-acetylgalactosamine (GalNAc) molecules may trigger the production of glycan-specific antibodies that cross-react with galactose-deficient IgA1 (Gd-IgA1) [110].

The presence of high levels of serum Gd-IgA1 is not sufficient to induce glomerular injury. It requires the formation of IgG or IgA1 autoantibodies that recognize the terminal GalNac of Gd-IgA1 as a neoepitope. These autoantibodies, mainly of the IgG isotype, share an unusual sequence in the variable region of their heavy chains that allows binding to galactose-deficient glycans of Gd-IgA1 [110]. Stimulation of NALT can also be an effect of dental caries. As was proven by Nagasawa et al. [106], smoking exacerbates dental caries, leading to activation of antibody production [111].

Other common mechanisms are alterations of the adaptive immune system. This might be due to the role of B and T cells and loss of immune tolerance [64]. It is well known that B cells are essential in humoral immunity to inhaled allergens [112]. Wu et al. [113] proposed a hypothetical model of B-cell involvement in the pathogenesis of MN. B cells recognize local antigens in lung tissue. B-cell surface TLRs and B-cell receptors (BCRs) that are activated during airway inflammation can recognize LPS and PLA2R, respectively, inducing circulating inflammatory responses that affect the phenotype and inhibitory function of regulatory B cells (Breg). Autoreactive B cells can participate in abnormal GC activity, and their entry to GC is supported by B-cell activating factor (BAFF). Autoreactive B cells then present antigens to T follicular helper cells (Tfh) at the light zone and T-B cell border of the GC, thus activating T-cell-dependent responses. Failed positive selection in the light zone and abnormal somatic hypermutation (SHM) and class-switch recombination (CSR) in the dark zone contribute to the formation of abnormal GCs in MN. Memory B cells then enter the circulation and long-lived plasma cells (LLPC) penetrate bone marrow. LLPCs in the bone marrow secrete autoantibodies while showing resistance to anti-CD20 drugs. Finally, autoantibodies bind to podocyte target antigens (e.g., PLA2R), causing glomerular damage and proteinuria [113]. This results in the statement that the lung acts as a critical site for immune activation in MN.

Antigen-presenting cells (APCs), for example, dendritic cells (DCs) in pulmonary tissue, when activated by smoking-induced inflammation, can cause loss of immune tolerance and induce epitope spreading, key steps in autoantibody development [64]. Moreover, DCs take up pathogens in tissues and present pathogen-derived antigens to T cells through MHC (major histocompatibility complexes), which activate naïve T cells. Cigarette smoke extract has been reported to modulate the function of DCs, so they induce Th2 differentiation essential for humoral responses. Also, nicotine has been shown to stimulate the Th2-inducing function of DCs [114]. Additionally, in MN, immune complex formation and deposition, and complement activation can occur.

### 5.3. Changes in Vasculature

Alternating often noted mechanisms of action in glomerulopathies due to cigarette smoking is known as hypertension. It has long been known that the nicotine content of cigarettes influences blood pressure and heart rate. It causes a rise in blood pressure by vasoconstriction and acceleration of the heart [115]. In vitro studies showed that cigarette smoke extract or isolated components such as nicotine decrease the availability of nitric oxide (NO), which is a powerful vasodilator [116]. In the RAA system, the role of angiotensin-converting enzyme (ACE) is to promote angiotensin I (Ang I) to generate angiotensin II (Ang II), where ACE2 is the specific negative regulatory member of the mechanism after RAA system activation. Yuan [117], in their research, found that expression levels of Ang II in the lung tissues of rats exposed to cigarettes were significantly increased, while ACE2 expression was reduced.

In IgAN, prolonged activation of the RAA system leads to hypertension that is associated with endothelial dysfunction and vascular smooth muscle proliferation [118,119,120]. Arteriolar wall thickening is a characteristic finding in nephrosclerosis that can lead to renal ischemia. Hypertension in renal arteries is also influenced by renal arteriolar hyalinosis and vasculature, promoting renal atherosclerosis [121]. Particularly, the inflammatory response is an essential component in the initiation and progression of atherosclerosis. Cigarette smoking stimulates the production of multiple pro-inflammatory mediators, especially C-reactive protein (CRP), IL-6, and TNF-α [116]. Progression of renal injury in MN appears to affect the renal vessels, which is associated with elevated plasma endothelin due to smoking.

## 6. Nicotine as a Main Compound Involved in Pathological Processes in the Kidneys

Among the thousands of compounds present in tobacco, nicotine is considered the most important factor responsible for a variety of biological effects that may play an important role in the pathogenesis of different conditions [118,122,123,124]. This one of the most stable and active components of cigarette smoke seems to play a key role in the onset and progression of proteinuria, diabetic nephropathy, and subsequently, CKD [120,125,126,127]. Several studies have proposed mechanisms that may cause nicotine-induced changes in the kidneys.

Hua et al. [128] proved that nicotine aggravates the severity of renal injury in a mouse DN model (db/db mice). Nicotine administered in the drinking water at a concentration of 100 μg/mL to db/db mice for 10 weeks significantly increased urinary protein excretion and caused glomerular hypertrophy. These changes were accompanied by significant increases in NADPH (nicotinamide adenine dinucleotide 2′-phosphate reduced tetrasodium salt hydrate) oxidase 4 (~30%) and increased nitrotyrosine expression. Jaimes et al. [37] confirmed that a similar increase in NADPH oxidase activity after nicotine exposure is observed in human podocytes. A similar effect was seen in mesangial cells [19,128], thus demonstrating that NADPH oxidase activity is a main source of ROS in nicotine-exposed kidney cells from both animals and humans. Increase in NADPH oxidase was accompanied not only by ROS generation but also by Akt phosphorylation in human mesangial cells [128]. Akt kinase is known for promoting kidney hypertrophy and extracellular matrix accumulation in an NADPH-dependent mechanism during diabetic nephropathy [129].

Jaimes et al. [130] showed that nicotine increases glomerular cell number in nephritic rats. It also significantly increased the expression of cortical fibronectin, which is a critical matrix component. In another study, Jaimes et al. [37] also confirmed that nicotine binds directly to podocytes via nicotinic acetylcholine receptors (nAChRs) to induce ROS generation and cyclooxygenase (COX-2) expression, increased CD36-mediated oxidized low-density lipoprotein (oxLDL) uptake, reduced podocyte maturity marker expression (i.e., synaptopodin), and induced their apoptosis, causing glomerular injury [37]. Podocyte apoptosis was related to the activation of mitogen-activated protein kinases (MAPKs) and oxidative stress [39]. Interaction of nicotine with proximal tubular cells also resulted in their apoptosis and epithelial–mesenchymal transition (EMT) [131,132].

Further studies confirmed that nAChR subunits are expressed in kidney mesangial cells and tubular cells [130,132]. In rats, the expression of α7-nAChR in the proximal and distal tubules was demonstrated [133]. In humans, high expression of nAChR α5, α6, α7, α10, and β4 was observed in podocytes [37]. At the same time, the expression of nAChR α3, α9, β2, and β3 was relatively lower, and the expression of nAChR α2 and α4 was barely detectable [118,134].

Apart from binding to nicotinic receptors, there is substantial evidence of the direct cytotoxic effects of nicotine and its metabolites on podocytes [38]. Most of the proteinuric diseases, including MCD, are associated with altered podocyte phenotype, reduction in their number, or effacement of foot processes [135]. Zarzecki et al. [136] showed that Sprague-Dawley rats exposed in utero to cigarette smoke extract have significantly reduced glomerular volume and podocyte number, which suggests that prenatal exposure to nicotine negatively influences glomerular development. Singh et al. [38] confirmed that nicotine activates NLRP inflammasome, reduces expression of podocyte maturity markers (i.e., podocin and nephrin), upregulates the expression of caspase 1 and IL-1β, and increases cellular permeability. In mesangial cells, nicotine activates TGF- and Wnt/-catenin pathways, which promote cell growth and production of extracellular matrix [130].

**Figure 2:**
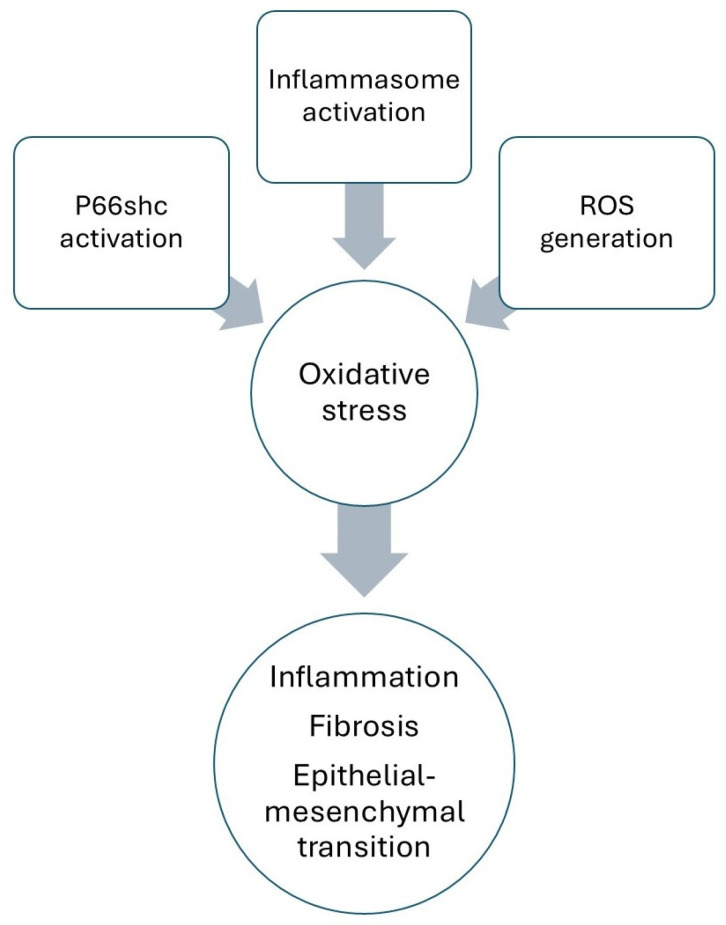
Role of oxidative stress in nicotine-induced kidney damage.

Nicotine increases ROS generation in a dose-dependent manner, where ROS has been incriminated for apoptosis in multiple instances [137]. In some studies, it has also been shown that podocytes, as highly differentiated epithelial cells, are more vulnerable to increased oxidative stress compared with mesangial cells. Nicotine increases ROS generation also in proximal tubule cells. Both active and passive forms of nicotine exposure enhance renal oxidative stress by increasing mitochondrial ROS production, transcriptional activation of the pro-apoptotic and prooxidant p66shc (oxidoreductase that produces ROS in a mitochondria-dependent manner) in renal proximal tubule cells [138], and NLRP3 inflammasome activation [38,139,140,141]. Oxidative stress steers the inflammatory cascade and renal fibrosis that stimulate chronic kidney injury and lead to ESRD (Figure 2) [38,140,142,143].

It was also demonstrated that nicotine decreases the expression of nephrin in podocytes [38]. Additionally, nicotine has been demonstrated to increase the proliferation of renal mesangial cells [19], proximal tubular epithelium, renal fibrosis, vimentin, and fibronectin production [130]. It was examined whether nicotine could affect apoptosis-related proteins, for example, caspase-3, which plays a key role in apoptosis. Studies confirmed that nicotine increases the expression of the cleaved form of caspase-3 and Bax (a pro-apoptotic protein) while decreasing Bcl-2 (an anti-apoptotic protein). Taking everything into consideration, we can say that nicotine increases podocyte apoptosis [39].

**Figure 3:**
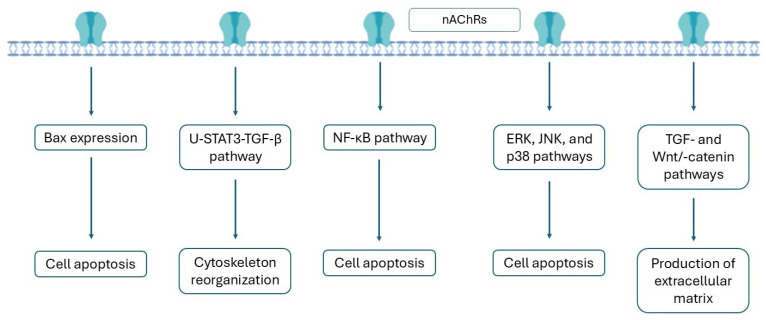
Signaling pathways activated in kidney cells after nicotine exposure.

Mitogen-activated protein kinases ERK, JNK, and p38 have also been implicated in nicotine-mediated podocyte damage and the progression of GN (Figure 3) [144,145]. Blockade of these kinases with their specific inhibitors significantly reduced nicotine-induced podocyte apoptosis. Kim et al. [145] demonstrated that nicotine-induced oxidative stress enhanced the phosphorylation of the ERK and JNK in renal proximal tubular cells, which stimulated the activation of the NF-κB signaling pathway and led to their apoptosis. In mice, chronic nicotine exposure stimulated the expression of the unphosphorylated signal transducer and activator of transcription-3 (U-STAT3) [132], which is associated with inflammation [146]. Increased expression of U-STAT3 caused transforming growth factor beta 1 (TGF-β1)-dependent transcriptional events that resulted in reorganization of the actin cytoskeleton and increased transcription of monocyte chemotactic protein-1 (MCP-1).

## 7. Discussion

In the literature, many reports suggest that cigarette smoking increases the risk of CKD, particularly CKD caused by hypertensive nephropathy and diabetic nephropathy. Clinical reports have demonstrated that smoking both worsens CKD and enhances proteinuria [130,147,148,149,150,151]. Therefore, it is possible that the contents of the tobacco smoke, especially nicotine, directly affect the podocytes. Molecular analysis and immunofluorescent staining revealed the expression of several nAChRs in podocytes and tubular cells. Nicotine was shown to decrease nephrin expression in podocytes, which indicates that it causes cell injury. It also enhances podocyte oxidative stress, resulting in their apoptosis. Nicotine-induced podocyte apoptosis was proven to be regulated by the activation of the kinase pathways (JNK, ERK, and p38). Even though nicotine mediates its effects mainly through the activation of muscle and neuronal nAChRs [130,134,152,153], it can also increase the proliferation of renal mesangial cells [19] and induce apoptosis of podocytes [144]. Inducing ROS generation contributes to the net oxidative stress imposed by cigarette smoking [131,134]. In previously reported studies, Nicotine has also been demonstrated to increase the production of ROS in cultured mesangial cells and stimulate their proliferation and fibronectin production [130].

**Figure 4:**
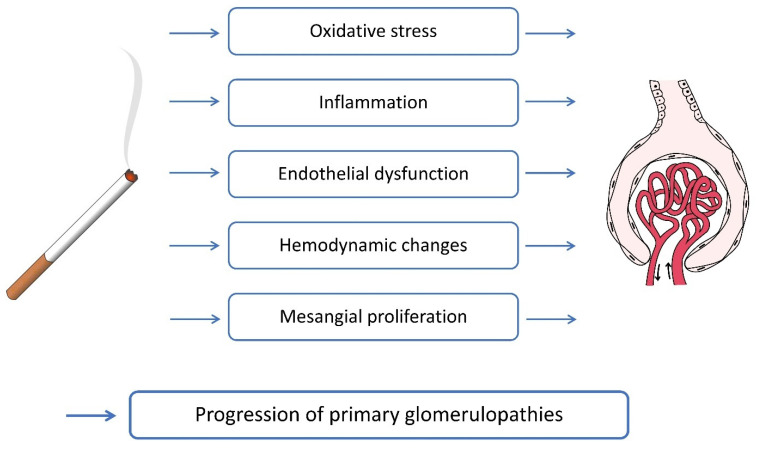
Summary of nicotine action on glomeruli in GN.

Many authors say that cigarette smoking disrupts kidney function, with a significant impact on health care outcomes and health care economics. In the last decade, several significant studies using animal models and human subjects demonstrated the role of cigarette smoking in the progression of chronic kidney disease (Figure 4). The effects of smoking, both active and passive, start at an early age and have long-lasting effects. The increasing prevalence of CKD demands stronger measures to halt the progression of the disease. This is particularly important as CKD patients are also at risk for significant cardiovascular diseases, in which smoking cessation provides cardiovascular and renal benefits [14].

Provenzano et al. [154] in their study emphasized that finding more factors impacting kidneys, in general, well-being would allow detection of patients who require stricter monitoring to more efficiently recognize progression of disease. Smoking not only affects the kidneys directly but, more importantly, worsens the course of other chronic diseases, such as cardiovascular disorders and proteinuria. Those morbidities negatively affect the functioning of the kidneys and, together with nicotine, cause CKD to progress more rapidly. Smoking is independently associated with a significantly increased risk for cardiovascular events and mortality in a large cohort of already diagnosed CKD patients. Moreover, any registered data suggests that current smokers are at higher risk for renal end-stage disease and cardiovascular events, compared to both former smokers and never-smokers, who were referred for the study. It highlights the importance of a holistic approach to the treatment of primary glomerulopathies. Patients are under increased risk already having other comorbidities, such as, as mentioned above, hypertension, and smoking might amplify these risks.

## 8. Conclusions

Tobacco consumption is associated with a broad spectrum of diseases, ranging from malignancies and cardiovascular disorders to chronic kidney disease and autoimmune conditions [155,156]. Notably, tobacco use remains the leading preventable cause of morbidity and mortality worldwide [157], accounting for more than 7 million deaths annually, including approximately 1.6 million deaths from secondhand smoke exposure [2]. Consequently, there is a pressing need to identify, within the population of tobacco users, those patients who are at potentially higher risk of tobacco-related diseases, including renal pathology. It has been proposed that exposure biomarkers with concentrations in biological fluids can vary according to the type and extent of tobacco use. Irrespective of the tobacco form, fluctuations are observed in the levels of pro-inflammatory cytokines (TNF-α, IL-1β, IL-6, IL-8, IL-17, IFN-γ), anti-inflammatory cytokines (IL-10, IL-4, IL-13), growth factors (TGF-β, VEGF, EGF, BDNF), selected biological active molecules (MMP-9, CRP, MCP-1), selected oxidative stress parameters (uric acid, glutathione, glutathione peroxidase, superoxide dismutase, malondialdehyde), and microplastics [1]. Future studies are necessary to explore the mechanisms of nicotine-induced renal injury. Large clinical trials evaluating the effects of smoking in patients with CKD, as well as the effects of smoking cessation on “renal health,” may help answer some significant questions, including measures to prevent the progression of kidney diseases.

## Figures and Tables

**Table 1:** Main factors related to smoking and their impact on glomeruli in GN.

Smoke-Related Factor	Potential Effects	Outcome	Glomerulopathy
Reactive oxygen species	Oxidative stress Aberrant glycosylation of IgA1 Epithelial cells loss Augmentation of pro-inflammatory cytokines	Stiffness of central vessels Rise in arterial pressure Tubular damage	IgA nephropathy Membranous nephropathy
Excessive antibody production	Activation of innate immunity leading to antigen presentation, and those present in dental caries	Damage to podocytes and tubular cells Damage to vessels	Minimal change disease Membranous nephropathy IgA nephropathy
Changes in vasculature	RAA system activation Renal arteriolar hyalinosis Renal atherosclerosis Elevated plasma endothelin levels	Endothelial dysfunction Vascular smooth muscle proliferation	IgA nephropathy Membranous nephropathy

## Data Availability

No new data were created or analyzed in this study. Data sharing is not applicable to this article.

## Abbreviations

The following abbreviations are used in this manuscript:

CKD	Chronic kidney disease
DN	Diabetic nephropathy
ESRD	End-stage renal disease
FSGS	Focal segmental glomerulosclerosis
GBM	Glomerular basement membrane
GN	Glomerulonephritis
IgAN	IgA nephropathy
MCD	Minimal change disease
MN	Membranous nephropathy
RAA	Renin–angiotensin–aldosterone
ROS	Reactive oxygen species
